# In-Cell DEER Spectroscopy of Nanodisc-Delivered Membrane Proteins in Living Cell Membranes

**DOI:** 10.1021/jacsau.4c00702

**Published:** 2024-09-24

**Authors:** Chu-Chun Cheng, Ruei-Fong Tsai, Che-Kai Lin, Kui-Thong Tan, Vidmantas Kalendra, Mantas Simenas, Chun-Wei Lin, Yun-Wei Chiang

**Affiliations:** † Department of Chemistry, National Tsing Hua University, Hsinchu 300-044, Taiwan; ‡ Faculty of Physics, Vilnius University, Sauletekio 3, LT-10257 Vilnius, Lithuania

**Keywords:** nanodisc, membrane protein, spin label, ESR/EPR, DEER/PELDOR

## Abstract

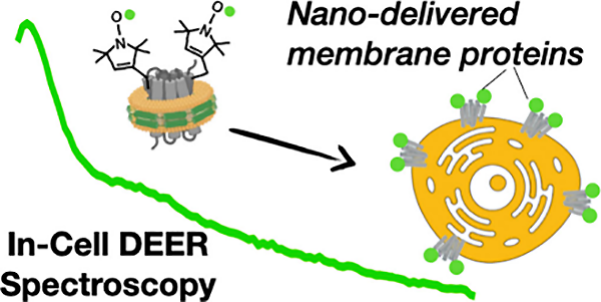

Membrane proteins are integral to numerous cellular processes, yet their conformational dynamics in native environments remains difficult to study. This study introduces a nanodelivery method using nanodiscs to transport spin-labeled membrane proteins into the membranes of living cells, enabling direct in-cell double electron–electron resonance (DEER) spectroscopy measurements. We investigated the membrane protein BsYetJ, incorporating spin labels at key positions to monitor conformational changes. Our findings demonstrate successful delivery and high-quality DEER data for BsYetJ in both Gram-negative *E. coli* and Gram-positive *B. subtilis* membranes. The delivered BsYetJ retains its ability to transport calcium ions. DEER analysis reveals distinct conformational states of BsYetJ in different membrane environments, highlighting the influence of lipid composition on the protein structure. This nanodelivery method overcomes traditional limitations, enabling the study of membrane proteins in more physiologically relevant conditions.

Membrane proteins exhibit a wide range of conformations, essential for activities such as channel operation, substance transport, and signal reception. Understanding these conformational shifts and equilibrium dynamics is crucial for mechanistically explaining their functions. Traditionally, the molecular structures of membrane proteins have been determined using techniques like X-ray crystallography, nuclear magnetic resonance (NMR), and cryo-electron microscopy (cryo-EM).^1,2^ These methods typically require isolating proteins from their natural environments using detergents and placing them in formats such as protein-detergent complexes, proteoliposomes, or lipid nanodiscs for specific measurements. However, this isolation can obscure critical cellular factors such as lipid composition, molecular interactions, and pH or ionic gradients, all of which can affect protein structure, function, and dynamics. Increasing evidence suggests that the native lipid environment is crucial for the proper folding, structure, and activity of membrane proteins.^3−5^ Consequently, capturing membrane protein conformations within their natural lipid environment has become a major goal in biochemistry and molecular biology.

Pulse dipolar spectroscopy techniques, such as pulse double electron–electron resonance (PELDOR or DEER), have become effective biophysical tools for measuring distances between two paramagnetic probes attached to a target protein.^6−9^ DEER enables nanometer-range distance measurements within proteins or protein complexes, allowing for the determination of structural conformations between functional states in equilibrium. Unlike traditional methods that depict proteins as static, single structures, DEER provides interspin distance distributions, capturing the conformational ensemble of a protein as it fluctuates within these states.^10−13^ This capability makes DEER a valuable complement to other structural biology tools. DEER allows for the observation of protein conformation changes between states and their responses to external perturbations such as pH, ion concentration, temperature, and ligands. By variation of these conditions, the same protein samples can be reused for DEER measurements, eliminating the need to prepare multiple batches of valuable protein samples.

DEER spectroscopy, when combined with nanodiscs, is particularly effective for studying the conformational changes of membrane proteins in lipid environments.^10,11,13^ However, the lipid content in nanodiscs is significantly less complex than that in native cellular membranes. The small size of nanodiscs prevents lipids from forming a thermodynamically stable ensemble phase similar to those in cells, potentially affecting protein conformations.^14,15^ Techniques to prepare native lipid nanodiscs are being developed.^16^ Additionally, in-cell DEER with in situ labeling to study membrane protein conformations is currently limited to outer-membrane proteins in Gram-negative bacteria, restricting the range of proteins that can be studied.^6,17^ The method involves the overexpressing target proteins, followed by spin labeling of engineered cysteines with nitroxide-based probes, either in *E. coli* or isolated outer membranes. It is challenging but has seen significant advancements.^18,19^ Other techniques, such as using spin-labeled nanobodies, are also being developed.^6,20^ Challenges like membrane protein aggregation in isolated membranes and low labeling efficiency result in poor signal-to-noise ratios (SNRs). Therefore, there is a growing need for methods that allow DEER measurements on membrane proteins directly within the native membrane of living cells.

To address these challenges, we propose using nanodiscs as a vehicle to introduce recombinant, spin-labeled membrane proteins into the membranes of living cells for DEER studies (Figure 1A). This nanodisc-based delivery technique, referred to as nanodelivery, was previously established for delivering cell-free synthesized membrane proteins into cells.^3,21^ However, it has not yet been applied to advance DEER spectroscopy for studying membrane proteins.

**Figure 1:**
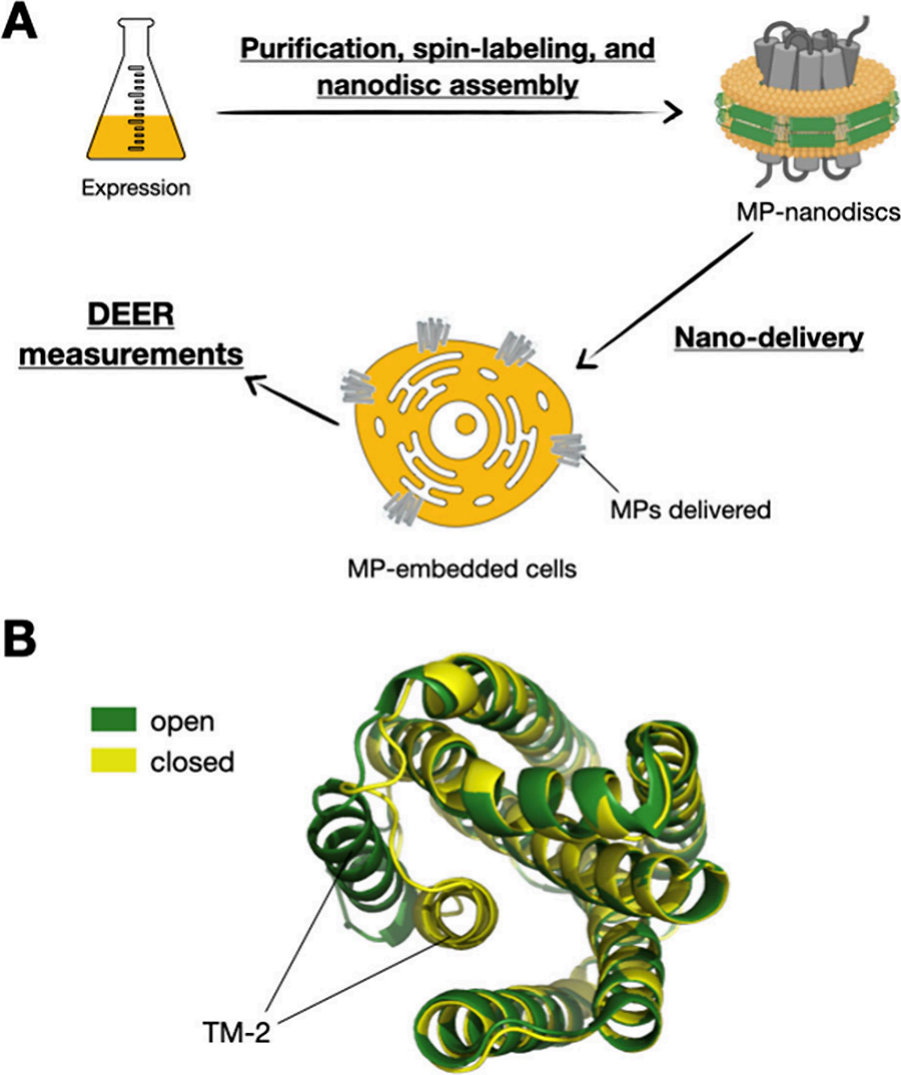
**Illustrations of the nanodelivery method and the BsYetJ models.** (**A**) The proposed approach involves purifying and spin-labeling membrane proteins (MPs) before integrating them into nanodiscs. Once delivered to cells using nanodiscs as a vehicle, DEER measurements can be performed directly in different cellular contexts. This strategy enhances the SNR of DEER data, facilitating the study of MPs in diverse cellular and lipid environments. (**B**) Crystal structure of BsYetJ showing the open and closed states (PDB codes: 4PGS, 4PGR), with a lateral movement in TM-2 being the primary difference between them.

By employing the nanodelivery method, our study aims to overcome the limitations of traditional approaches by enabling direct DEER measurements on proteins transported into various cellular environments. Our approach involves purifying and spin-labeling membrane proteins before their integration into nanodiscs, allowing for various modifications of the target proteins (Figure 1A). Once these spin-labeled proteins are delivered to the cells using nanodiscs as a vehicle, DEER measurements can be performed directly within different cellular contexts. This strategy underscores the versatility of nanodiscs in delivering chemically modified and functional proteins to cells, facilitating DEER measurements. This controlled delivery is expected to enhance the SNR of DEER data, enabling the study of membrane proteins in diverse cellular and lipid environments.

The membrane protein to be used in this study is BsYetJ, a bacterial homologue of the human TMBIM 6 membrane protein from *Bacillus subtilis*.^11,22,23^ BsYetJ is a 7-helix transmembrane (TM) protein composed of 214 residues (approximately 24 kDa). Previously, detergent-solubilized crystal structures of BsYetJ have shown two distinct conformational states (Figure 1B), with a lateral movement in TM-2 being the primary difference between the closed and open states.^23^ In prior work, we demonstrated that BsYetJ embedded in lipid nanodiscs exhibits conformational changes observable by DEER measurements.^11^ This study aims to extend those observations by utilizing nanodelivery to embed spin-labeled BsYetJ directly into native cellular membranes, allowing for a more accurate representation of its behavior in various cellular environments.

To evaluate the efficiency of transferring BsYetJ from nanodiscs to the membranes of *E. coli* cells, we prepared a cysteine variant of BsYetJ at solvent-exposed site 33C and attached an Alexa-647 fluorophore to this site as an indicator of protein localization. The fluorophore-labeled BsYetJ (hereafter referred to as Fluo-BsYetJ) was then incorporated into nanodiscs and incubated with *E. coli* cells at 37 °C for various durations (0.5–2 h; see Figures S1–S2 and Supporting Information Methods for details). Protein transfer was halted by centrifugation, and the resulting cell pellets were washed with a buffer and then collected for fluorescence quantification.

We observed an increase in fluorescence intensity over the incubation period, indicating the progressive delivery of Fluo-BsYetJ from nanodiscs to *E. coli* membranes (Figure 2A). Significant fluorescence intensity in the washed pellet was detected after just 1 h of incubation, suggesting a fast and efficient nanodelivery process. Comparing the fluorescence intensities for different incubation times with the control (a solution containing the same input amount of Fluo-BsYetJ-loaded nanodiscs without *E. coli* cells) showed that approximately 40% of Fluo-BsYetJ was transferred to the *E. coli* cell pellets within 1 h. The successful delivery of Fluo-BsYetJ to *E. coli* cells was also confirmed via confocal microscopy (Figure 2B). The images showed significant overlap between Fluo-BsYetJ and a small plasma membrane-targeted dye (PM-1)^24^ in the merged confocal microscopy image, verifying the localization of Fluo-BsYetJ on the *E. coli* membranes.

**Figure 2:**
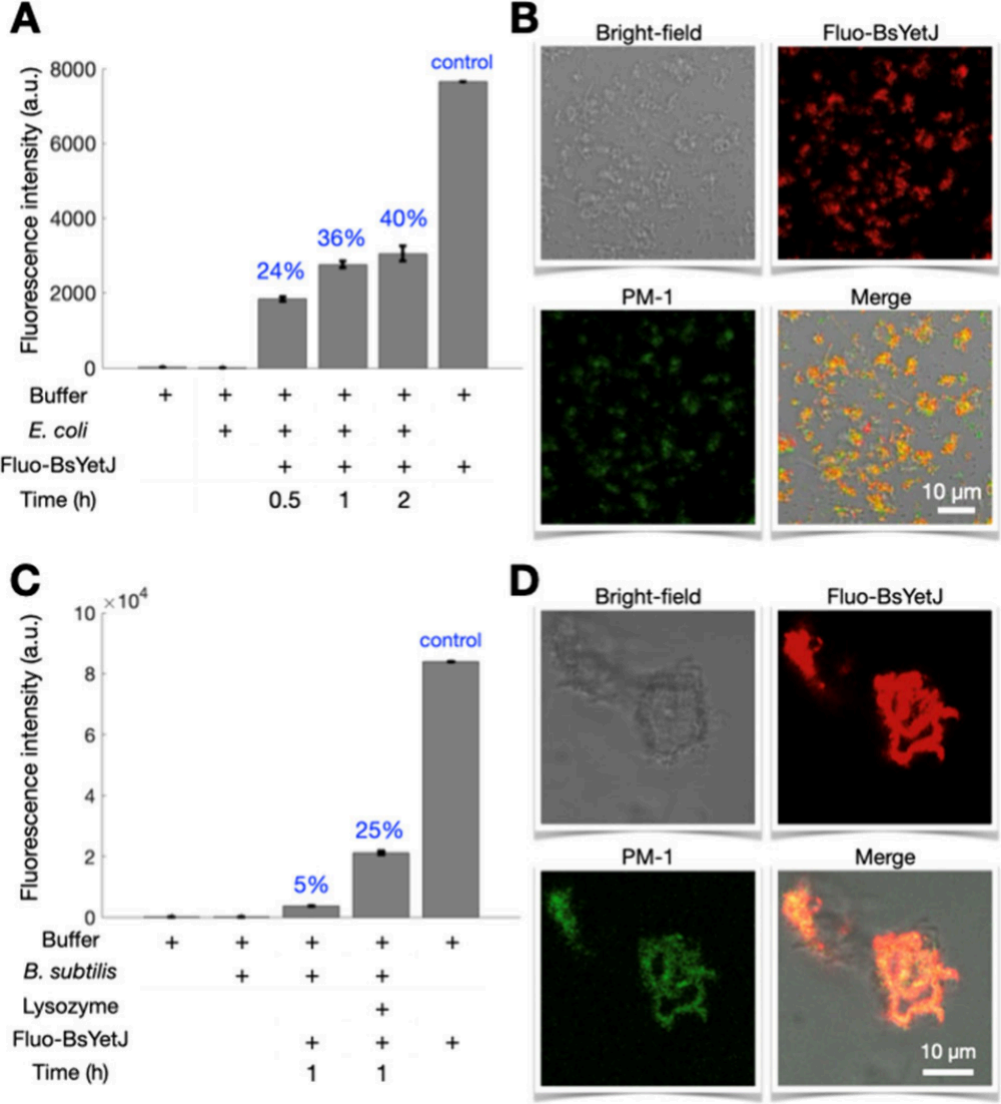
**Efficiency of transporting BsYetJ into the membranes of living cells.** (**A**) Fluorescence intensity of Fluo-BsYetJ (Alexa-647) in various incubation conditions. Approximately 40% of the input Fluo-BsYetJ was tranferred to *E. coli* membranes within 1 h. Transferring rates are noted in the plot. (**B**) Confocal images of *E. coli* cells after 1-h incubation with Fluo-BsYetJ-loaded nanodiscs. (**C**) Fluorescence intensity of Fluo-BsYetJ (Alexa-568) in various incubation conditions. Over 25% of the input Fluo-BsYetJ was transferred to *B. subtilis* protoplasts within 1 h. (**D**) Confocal images of *B. subtilis* protoplasts after 1-h incubation with Fluo-BsYetJ-loaded nanodiscs.

In addition to *E. coli*, a Gram-negative bacterium, we also demonstrated the nanodelivery method on *B. subtilis*, a Gram-positive bacterium. For this study, we used the fluorescence probe Alexa-568 to prepare Fluo-BsYetJ. The cytoplasmic membrane of Gram-positive *B. subtilis* is protected by a peptidoglycan cell wall, which significantly reduces membrane accessibility and hinders protein delivery (Figure 2C). Consequently, very little Fluo-BsYetJ was transferred to *B. subtilis*, as indicated by the weak fluorescence intensity in the pellets. To enhance delivery, we treated *B. subtilis* cells with lysozyme to degrade the cell wall, resulting in *B. subtilis* protoplasts (see Supporting Information Methods). These protoplasts were then incubated with Fluo-BsYetJ-loaded nanodiscs for 1 h at 37 °C to facilitate the transfer of Fluo-BsYetJ. The viability of the *B. subtilis* protoplasts was confirmed using agar plates (Figure S1C). Notably, we observed a significant increase in fluorescence intensity from the protoplast pellets, nearly six times higher than in cells not treated with lysozyme. This corresponds to a successful delivery of 25% of the input Fluo-BsYetJ. We confirmed that the delivered BsYetJ retains its ability to transport Ca^2+^ ions across membranes (Figure S3).

These results indicate a remarkable improvement in the BsYetJ transfer efficiency to *B. subtilis*, making DEER measurements on Gram-positive bacteria highly feasible. The morphology of the protoplasts and the localization of BsYetJ were confirmed by confocal microscopy (Figure 2D), which clearly showed the colocalization of Fluo-BsYetJ and PM-1 dyes.

Next, we performed DEER measurements on two doubly spin-labeled BsYetJ proteins after transferring them from nanodiscs to two different types of cells: *E. coli* and *B. subtilis*. The two variants, denoted as 33/55R1 and 22/184R1 (Figure 3A), have spin labels at positions spanning important transmembrane helices (TM-1, TM-2, and TM-7), covering periplasmic, cytoplasmic, and transmembrane domains. After incubating the spin-labeled BsYetJ-loaded nanodiscs with the respective cells at 37 °C for 1 h, DEER measurements were conducted at 80 K. CW-ESR was used to determine the optimal incubation time of 1 h (Figure S4). Each DEER measurement was completed efficiently within 20 min, enabled by an ESR cryoprobe equipped with a cryogenic microwave preamplifier.^25^ According to our protocol, approximately 5–10 nmol of spin-labeled BsYetJ was delivered to cells in the quartz tube within the DEER cavity (10–40 μL of effective volume of sample). This protein concentration (approximately 250 μM) is more than sufficient for obtaining high SNR DEER data. Details of the protocol are provided in the Supporting Information Methods.

**Figure 3:**
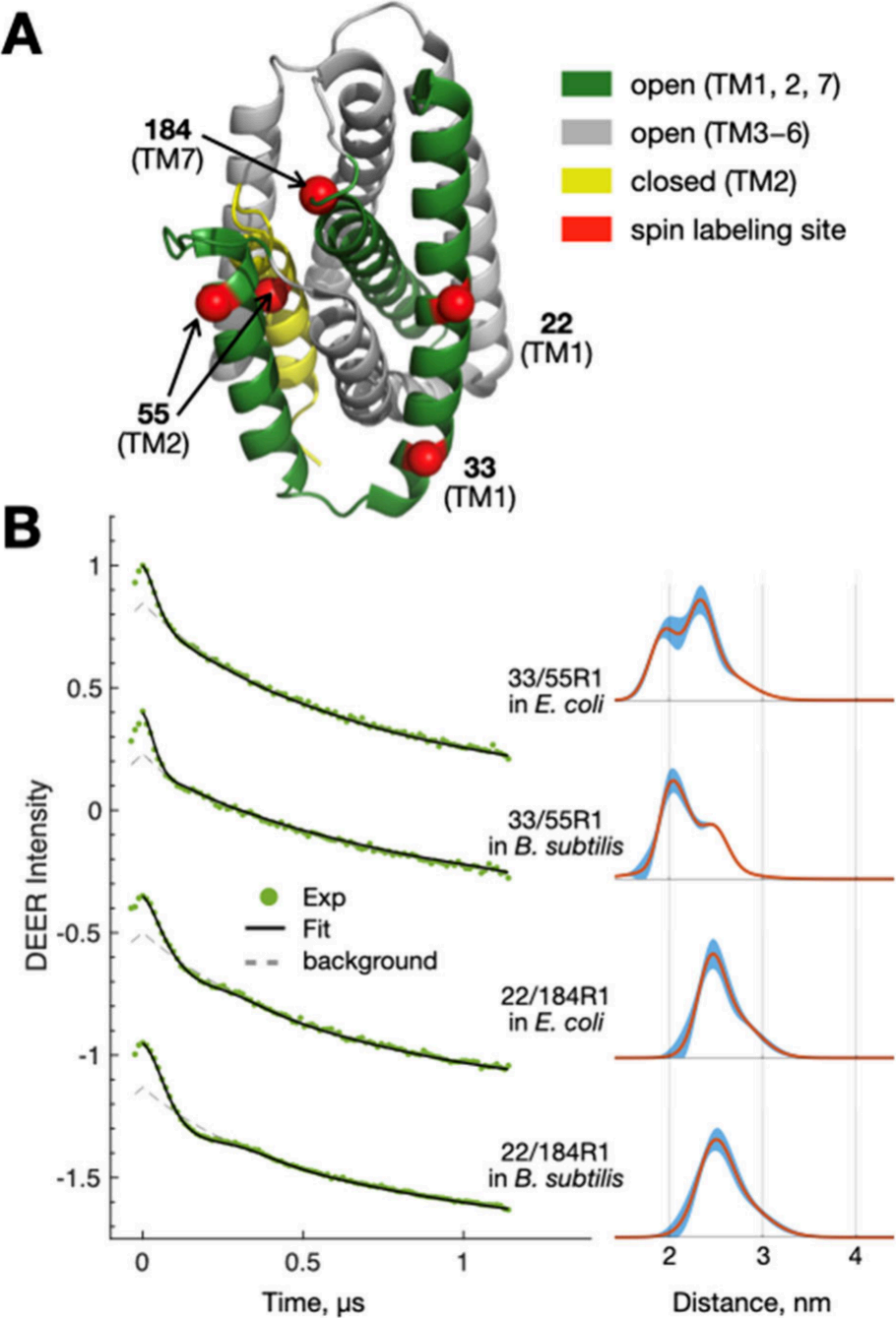
**DEER measurements.** (**A**) Cartoon models of BsYetJ closed and open states displaying the spin-labeling sites studied. (**B**) Left: Raw experimental DEER traces and model fits to the data. Right: Interspin distance distributions obtained from the model fits. Shading around the line indicates uncertainty bounds (2 times of standard deviation).

High-quality DEER signals were obtained for all samples, characterized by clear and distinct modulation depths accounting for 10–15% of the signal intensity (Figure 3B; also Figure S5 for phase memory time). The DEER data were then analyzed to yield interspin distance distributions (Figures 3B and S6).^26−29^ The results for 33/55R1 showed bimodal-like distance distributions, with two major peaks at 2.0 and 2.4 nm, corresponding to respective populations of 33% and 67% for *E. coli* and 57% and 43% for *B. subtilis*. This indicates that 33/55R1 exists in two conformational states within the cell membranes, consistent with previous DEER results of 33/55R1 measured in 100% POPC lipid nanodiscs, where the shorter and longer distances were identified as the closed and open states of BsYetJ.^11^ However, previous results indicated that although the populations of the two states change with pH conditions, the closed state remains dominant within the pH range (from pH 6 to 8) studied. The present study shows that the open state is dominant in *E. coli* membranes, while the closed state is dominant in *B. subtilis* membranes, highlighting significant differences between the two membrane environments. This suggests that the structural conformations of BsYetJ depend on the lipid environment (Figure S7), underscoring the value of our nanodelivery method, which allows the convenient delivery of membrane proteins to various cell membranes for studying structure–function relationships.

We also collected DEER data for 22/184R1 delivered to *E. coli* and *B. subtilis* membranes. The analysis of the DEER data showed a homogeneous distance distribution with a dominant peak at 2.5 nm for 22/184R1 in both environments, indicating that BsYetJ 22/184R1 remains in a single structural conformation. This result is consistent with our expectations, as previous studies based on detergent-solubilized crystals of BsYetJ and DEER measurements of BsYetJ-embedded nanodiscs have indicated that helices TM-1 and TM-7, where 22R1 and 184R1 are located, do not change between the open and closed states.^11,23^

In conclusion, our study demonstrates the effectiveness of the nanodelivery method for introducing spin-labeled membrane proteins into the membranes of living cells for DEER spectroscopy. Utilizing nanodiscs, we successfully delivered recombinant BsYetJ proteins to both Gram-negative *E. coli* and Gram-positive *B. subtilis* cells, with the delivered BsYetJ retaining its calcium ion transport functionality. This approach allowed for efficient DEER measurements in diverse cellular environments, effectively overcoming the limitations of traditional methods. The DEER data revealed significant differences in the conformational states of BsYetJ between the two types of membranes, highlighting the critical role of the lipid environment in determining the protein structure. The successful delivery and subsequent DEER analysis of BsYetJ in *E. coli* and *B. subtilis* underscore the versatility and potential of the nanodelivery method for studying membrane proteins across different cell membranes. Our findings suggest that Gram-positive cells like *B. subtilis*, which have a single membrane, are particularly well-suited for future applications of this method, as opposed to Gram-negative cells with two membrane layers, which may complicate the results. This insight is especially valuable because most mammalian cells, similar to other eukaryotic cells, possess only one lipid bilayer forming the plasma membrane. This advancement positions DEER spectroscopy as a powerful tool for elucidating the structural conformations and dynamic behaviors of membrane proteins in biologically relevant environments.

## Data Availability

All data needed to evaluate the conclusions in the paper are present in the paper and/or the Supporting Information.

## Supporting Information Available

Materials and Methods. Figure S1 shows labeling efficiency and viability tests. Figure S2 presents the results with Fluo-MSP and FPLC. Figure S3 shows the measurements of calcium flux activity for the delivered BsYetJ. Figure S4 shows CW-ESR results. Figure S5 presents the results of the ESE measurements. Figures S6 and S7 show complementary DEER results (PDF)
