# Supplementary material for: In-Cell DEER Spectroscopy of Nanodisc-Delivered Membrane Proteins in Living Cell Membranes
Source: JACS Au. 2024 Sep 24;4(10):3766–70. doi: 10.1021/jacsau.4c00702 (PMC11522923; doi:10.1021/jacsau.4c00702)
Supplement: Supplementary file 1 — au4c00702_si_001.pdf [file au4c00702_si_001.pdf]

Supplementary Materials for

In-Cell DEER Spectroscopy of Nanodisc-Delivered Membrane Proteins in Living Cell Membranes

Chu-Chun Cheng,[a] Ruei-Fong Tsai,[a] Che-Kai Lin,[a] Kui-Thong Tan,[a] Vidmantas Kalendra,[b] Mantas Simenas,[b] Chun-Wei Lin,*[a] and Yun-Wei Chiang*[a]

[a]Department of Chemistry, National Tsing Hua University, Hsinchu 300-044, Taiwan

[b]Faculty of Physics, Vilnius University, Sauletekio 3, LT-10257 Vilnius, Lithuania

*Corresponding emails: chunweilin@mx.nthu.edu.tw and ywchiang@mx.nthu.edu.tw

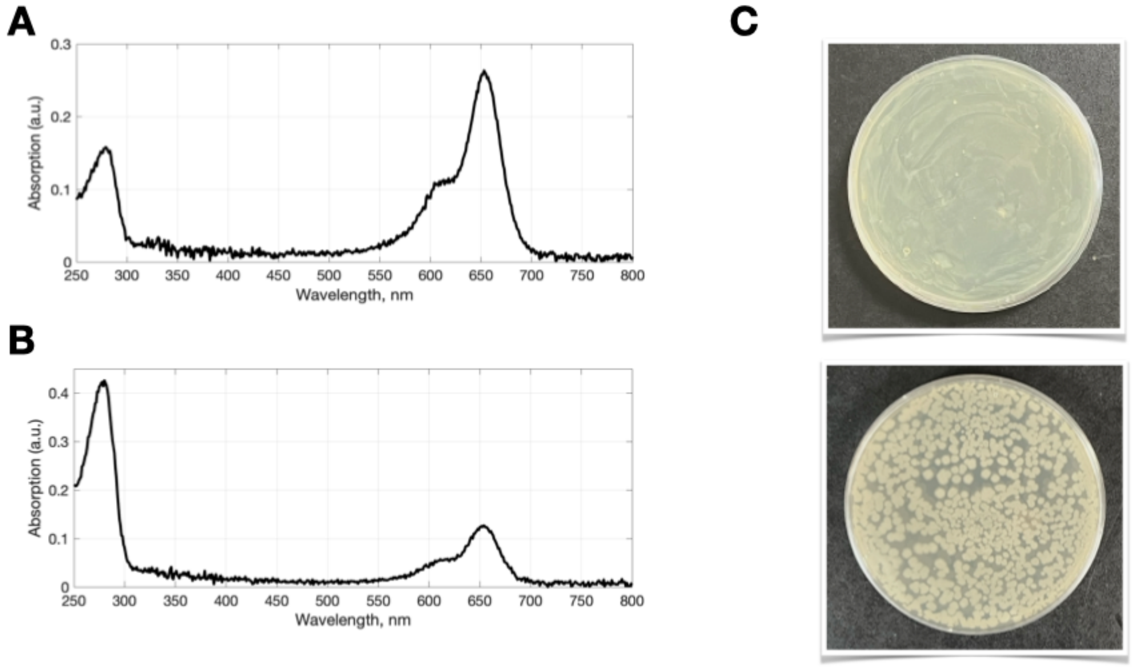

Figure S1. Labeling efficiency and cell viability of the nano-delivery method. (**A**) UV-Vis absorption spectrum of DDM-solubilized Fluo-BsYetJ (Alexa-647). By comparing the absorption at 651 nm (A_{651}= 0.258; extinction coefficients 265000 cm^{-1} M^{-1}) to that at 280 nm (A_{280}= 0.148; extinction coefficients 24410 cm^{-1} M^{-1}), corresponding to the input of BsYetJ proteins, we obtained concentrations of 0.97 µM and 5.96 µM for Alexa-647 and BsYetJ, respectively. Thus, we calculated a labeling efficiency of 16.2% for the Alexa-647 probe attached to the cysteine variant BsYetJ 33C under the experimental conditions provided in the SI Methods. (**B**) UV absorption spectrum of the sample solution after purification with size-exclusion FPLC chromatography. The purified solution contains both empty nanodiscs (i.e., containing no BsYetJ) and BsYetJ-loaded nanodiscs, which include membrane scaffold proteins (MSP) that maintain the nanodiscs' shape. Both MSP and BsYetJ contribute to the UV absorption at 280 nm. Combined with the Alexa-647 absorption peak at 651 nm from Fluo-BsYetJ, our results indicate that the incorporation efficiency of DDM-solubilized BsYetJ into nanodiscs is approximately 34%. (**C**) Agar plates (Upper: *E. coli*; Lower: *B. subtilis*). **Upper:** The viability of *E. coli* cells containing BsYetJ-33R1 after CW-ESR measurements was assessed by plating them on agar plates supplemented with kanamycin and chloramphenicol and incubating at 37 °C for 20 hours. The survival of the cells under antibiotic conditions indicates that the transformed plasmid remained functional, and cellular activities

necessary to produce proteins for antibiotic resistance were maintained. Combined with the time-dependent decrease in peak intensity of the ESR spectra (Fig. S4), these results further confirm the insertion of BsYetJ into the *E. coli* outer membranes, exposing the MTSSL label to the reducing environment generated by normal cellular activities. **Lower:** The viability of *B. subtilis* protoplasts containing BsYetJ-33R1 after DEER measurements was assessed by plating them on agar plates supplemented with chloramphenicol and incubating at 37 °C for 20 hours. The robust growth of the protoplasts indicated that the *B. subtilis* cells remained alive throughout the entire sample preparation process, including lysozyme treatment and BsYetJ delivery. This supports the assertion of the present study that we are performing in-cell DEER spectroscopy of nanodisc-delivered membrane proteins in living cell membranes.

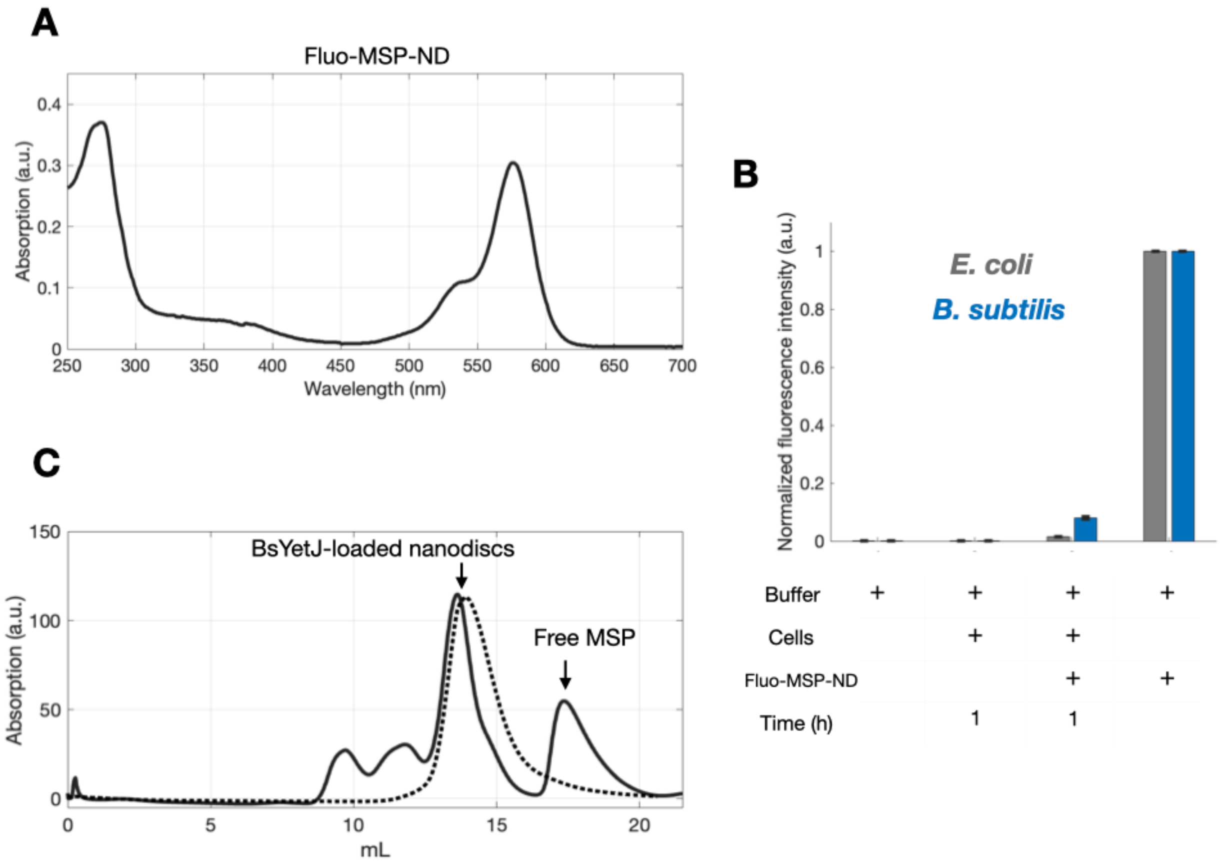

Figure S2. Assessment of nanodisc integrity and MSP retention post-delivery of BsYetJ to bacterial cells. To investigate the location and state of MSP and BsYetJ-loaded nanodiscs after delivery, we prepared a BsYetJ-loaded nanodiscs sample using Alexa-568-labeled MSP (referred to as Fluo-MSP). Hereafter, the BsYetJ-loaded nanodisc made with Fluo-MSP is referred to as Fluo-MSP-ND. Fluo-MSP was prepared with a cysteine variant of MSP, Q107C, previously shown to form lipid nanodiscs as efficiently as native MSP.[30] Fluorophore labeling was performed by incubating purified MSP Q107C with a 5-fold molar excess of Alexa Fluor 568 C5 maleimide (Thermo Scientific) in the dark for 15 hours at 4 °C. Unbound fluorophore was then removed by buffer exchange, and the Fluo-MSP was concentrated and used to prepare BsYetJ-loaded nanodiscs. (**A**) UV-Vis absorption spectrum of Fluo-MSP-ND (i.e., BsYetJ-loaded nanodisc made with Fluo-MSP). (**B**) Fluorescence intensity at 610 nm upon excitation at 575 nm of Fluo-MSP-ND under various incubation conditions. Sample preparation for fluorescence quantification was the same as in the nano-delivery efficiency tests, except Fluo-MSP (Alexa-568) was used here. Our results indicate that only 1.5% and 7% of the input Fluo-MSP were retained in *E. coli* cells and *B. subtilis* protoplasts, respectively. These fractions are negligible compared to the Fluo-BsYetJ retention,

which ranged from 25-40% (Figs. 2A and 2C). (**C**) Size exclusion chromatography (SEC) of crude BsYetJ-loaded nanodiscs supplemented with purified MSP (solid line) and the supernatant collected after 1-hour incubation of *E. coli* cells with BsYetJ-loaded nanodiscs (dotted line). The SEC results show successful separation of BsYetJ-loaded nanodiscs from free MSP, with a peak-to-peak retention volume difference of nearly 4 mL. The supernatant SEC profile (dotted line) is dominated by a single peak at nearly the same position as the BsYetJ-loaded nanodiscs, with no free MSP detected. The minor broadening of the supernatant nanodisc peak indicates decreased structural homogeneity of nanodiscs after BsYetJ transfer to cells. In conclusion, combined with fluorescence assay results, this indicates that most nanodiscs maintain their structure and diffuse away from the cells after BsYetJ delivery, leaving only a very small amount of MSP in the cells.

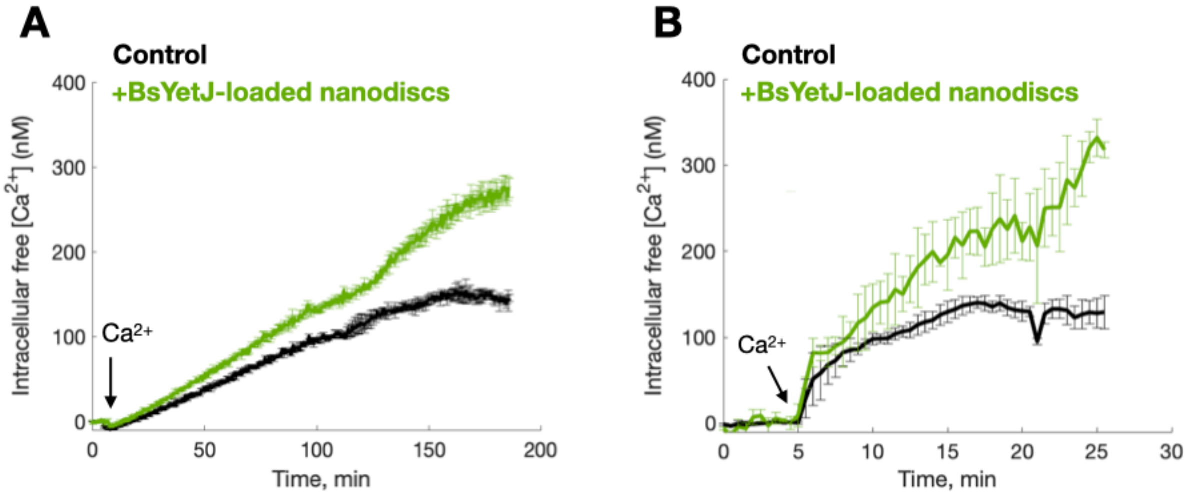

Figure S3. Calcium flux assay demonstrating the functional activity of delivered BsYetJ in bacterial membranes. Calcium flux into **(A)** *E. coli* cells and **(B)** *B. subtilis* protoplasts after 1 h incubation with BsYetJ-loaded nanodiscs. Cells not treated with BsYetJ-loaded nanodiscs served as controls. Error bars represent ±SD (n = 3 experiments). Calcium indicator Fluo-8/AM was used as detailed in the SI Methods. The arrow indicates the addition of 10 mM $CaCl_2$. Parallel to the structural study performed by DEER spectroscopy, we measured the calcium channel activity of BsYetJ post-delivery to verify its location and folding state in the bacteria. For both *E. coli* and *B. subtilis*, we observed robust and sustained calcium influx in cells treated with BsYetJ-loaded nanodiscs. Since calcium was added externally, the observed increases in fluorescence intensity indicate that the majority of the delivered BsYetJ remained in the membranes to facilitate calcium transport, rather than being trapped in the periplasm or internalized into cellular compartments like endosomes. Due to the different nature of lipids between *E. coli* cells (with two layers of membranes) and *B. subtilis* protoplasts (with only one membrane), we observed that calcium accumulation and flux rates in *E. coli* cells were much slower than those in *B. subtilis* protoplasts. Together with the delivery efficiency tests (Figs. 2A, 2C) and DEER results (Fig. 3B), these findings indicate that our nanodiscs efficiently deliver large quantities of BsYetJ to cellular membranes while preserving its structure and functionality.

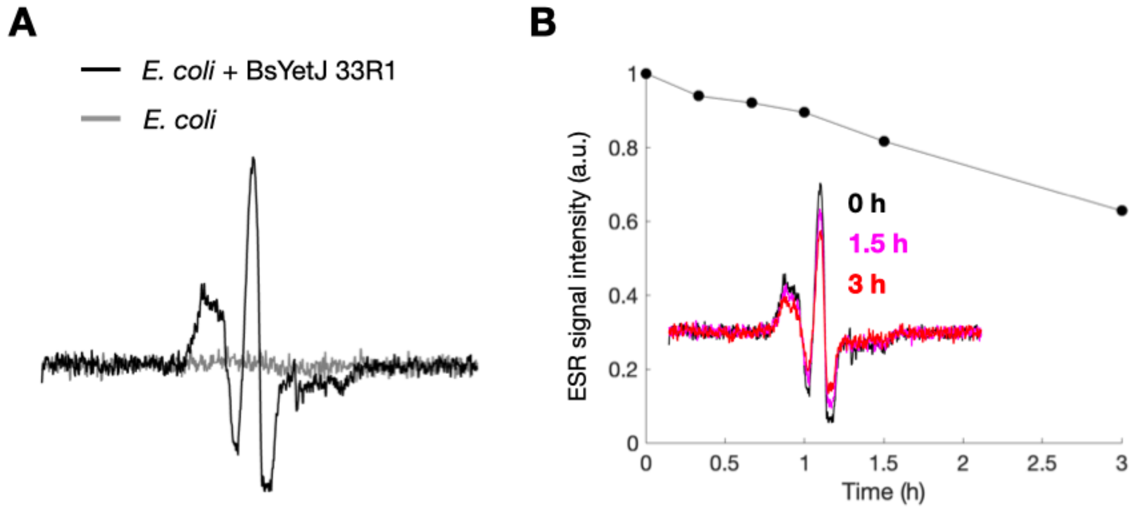

Figure S4. Evaluation of nitroxide reduction in the cellular environment. (**A**) CW-ESR spectra recorded at room temperature for two ESR tubes containing the same amount of *E. coli* pellet samples after 1 hour of incubation. One tube containing nanodisc-delivered spin-labeled BsYetJ 33R1 exhibits a typical CW-ESR spectrum of spin-labeled proteins, characterized by a slow-motional anisotropic spectral lineshape. This result confirms the delivery of spin-labeled BsYetJ to the cell membranes in the pellets. (**B**) Plot of central peak intensity of ESR spectra recorded over 3 hours for the sample of 1-hour incubation of *E. coli* with BsYetJ-33R1-loaded nanodiscs. Based on the intensity of the ESR spectral peak, we observed a reduction in the ESR spectra in the *E. coli* cellular environment to 90% of the initial intensity within 1 hour and 60% within 3 hours. The longer the incubation, the weaker the ESR intensity due to the reduction of nitroxide radicals in the cellular environment. Therefore, our protocol dictates that after 1 hour of incubation of cells with spin-labeled BsYetJ-loaded nanodiscs, the reaction should be quenched by centrifugation, and the sample should be frozen and sent for DEER measurements. Considering the finding (Fig. 2) that over 40% of BsYetJ was transferred from nanodiscs to *E. coli* membranes within 1 hour of incubation, we determined that the optimal incubation time in this nano-delivery method is 1 hour. Besides, we want to highlight that the CW-ESR spectra remained largely consistent, exhibiting a slow-motional lineshape throughout the incubation period. There was no indication of sharp spectra that would suggest the presence of free spin probes. This provides further evidence that our established protocol effectively minimizes the unwanted release of spin probes.

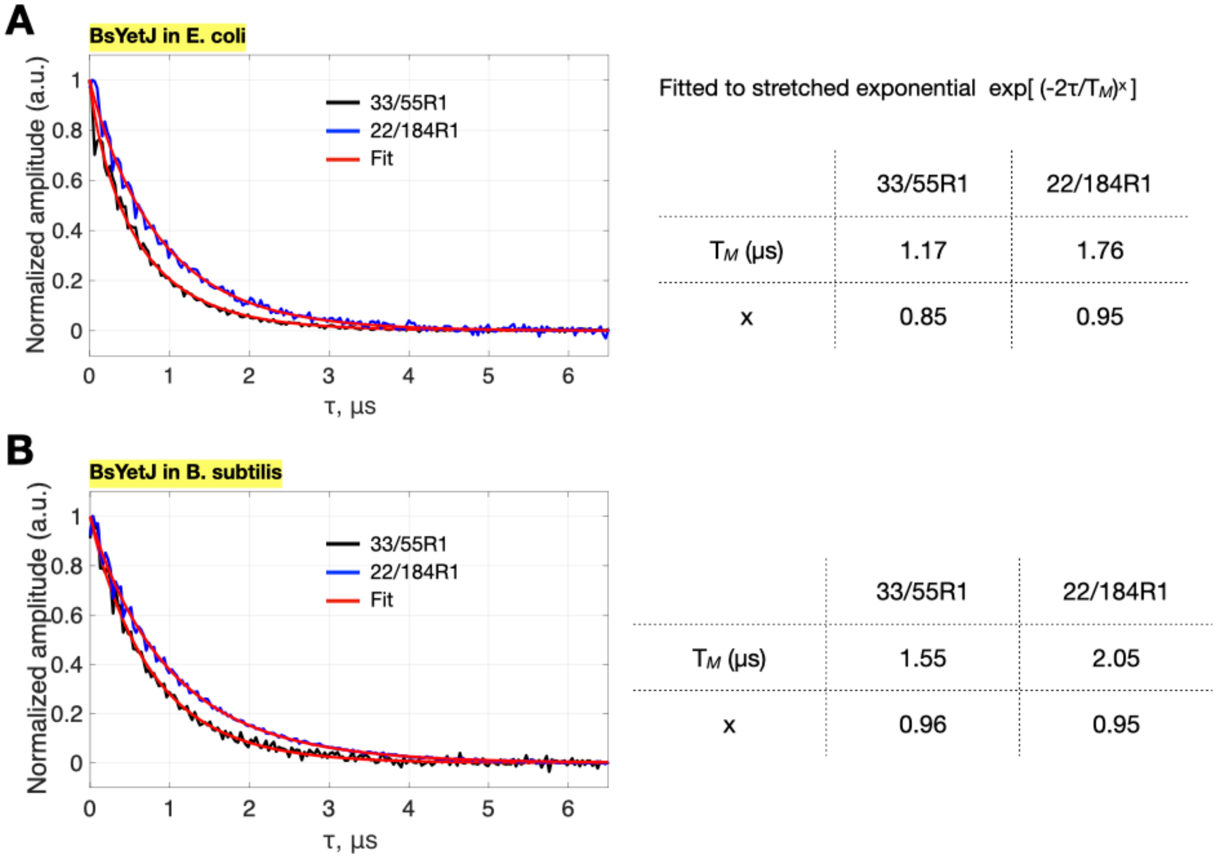

Figure S5. Measurements of phase-memory time with the ESE experiment. Theoretical fits (red lines) to the ESE experimental data of spin-labeled BsYetJ in two different cell membranes: (**A**) *E. coli* and (**B**) *B. subtilis*. The fits use a stretched exponential function as shown in the figure. The obtained values of phase-memory time (T_M) and the stretching exponent (x) are displayed in the plot. The T_M values are sufficiently long (over 1 μs) to ensure reliable DEER measurements.

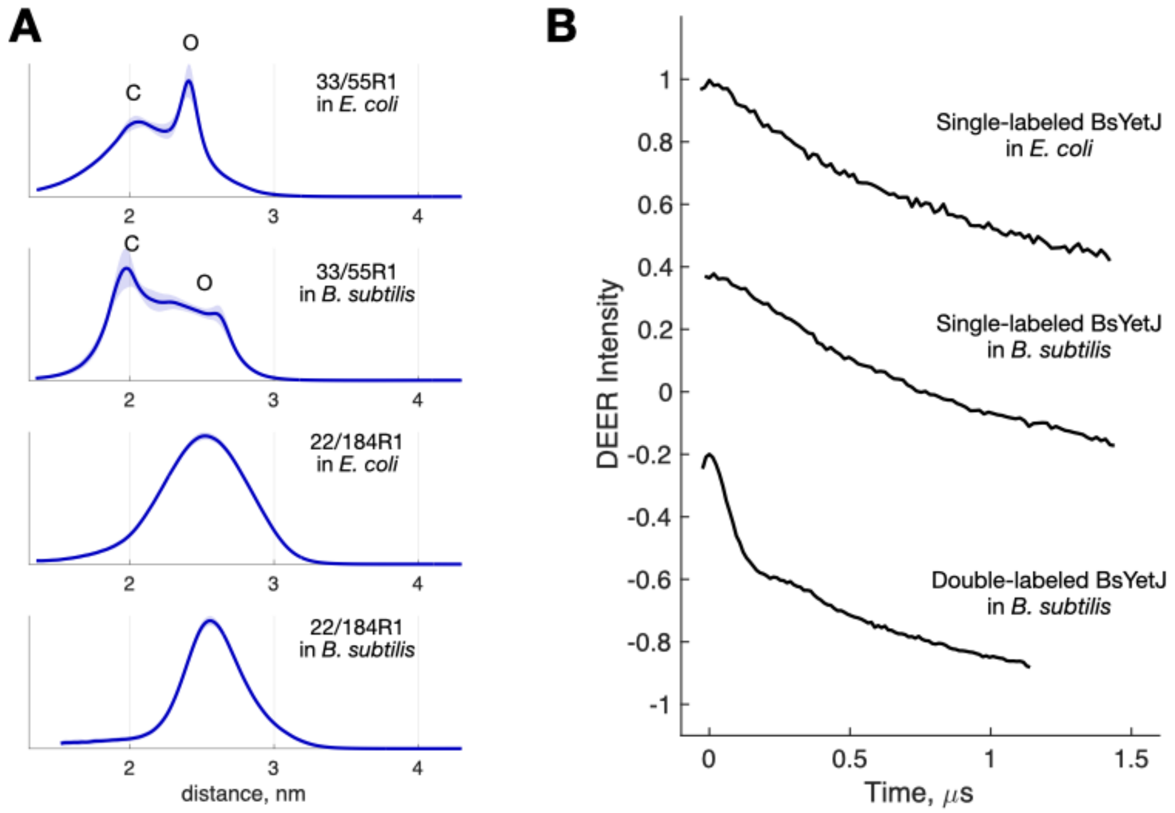

Figure S6. Supplementary DEER analysis and measurements. (**A**) Analysis results of the DEER measurements using DEERnet, a deep neural network for DEER data analysis. The DEERnet results align well with the model-based methods (Fig. 3), showing that the 22/184R1 pair exhibits homogeneous distance distributions in both environments (*E. coli* and *B. subtilis*). In contrast, the 33/55R1 pair displays bimodal-like distance distributions, with the open state (longer distance, denoted as O) and closed state (shorter distance, denoted as C) being dominant in *E. coli* and *B. subtilis*, respectively. The agreement between DEERnet and model-based results provides an independent validation, enhancing confidence in this ill-posed data analysis. Shaded areas represent the 95% confidence interval. (**B**) Raw experimental DEER data for singly labeled BsYetJ in two different environments (*E. coli* and *B. subtilis*). The DEER traces exhibit a homogeneous decay function with no indication of modulation depth, suggesting that the singly labeled BsYetJ proteins are not clustering to form specific oligomers and are homogeneously distributed in the studied cells. For comparison, the DEER trace of doubly labeled BsYetJ (22/184R1) in *B. subtilis* cells is shown, illustrating that its background decay matches the decays of the singly labeled

DEER traces. Given that the protein concentrations in all three samples are approximately the same, the similarity in decay patterns is expected. These findings strongly support the absence of BsYetJ oligomer or cluster formation following delivery to the cells.

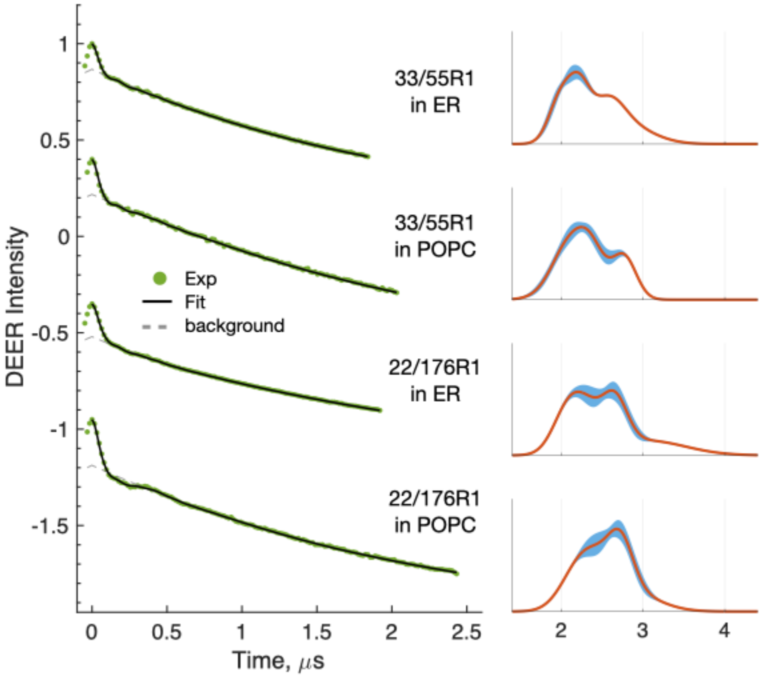

Figure S7. Supplementary DEER analysis of BsYetJ in different lipid nanodiscs. To further explore the in-cell DEER observations and investigate the lipid-dependent conformational dynamics of BsYetJ, we conducted DEER measurements on doubly spin-labeled BsYetJ variants reconstituted in nanodiscs with two distinct lipid compositions: one composed entirely of POPC lipids, and the other mimicking the endoplasmic reticulum (ER) with a lipid mixture of 61% POPC, 20% POPE, 11% POPI, and 8% cholesterol (molar ratios). Shown on the left are the experimental DEER traces and model fits to the data. Shown on the right are the corresponding distance distributions. For the 33/55R1 variant, the closed conformation (approximately 2 nm) was predominant in both POPC and ER nanodiscs, with a slightly higher presence of the open conformation (approximately 2.6 nm) in the POPC nanodiscs. However, a significant shift in distance distribution was observed for the 22/176R1 variant (TM1/TM6) between the two lipid environments. These findings confirm that BsYetJ exhibits lipid-dependent conformational equilibrium. These supplementary DEER measurements underscore the significance and versatility of the nano-delivery method presented in this study, demonstrating its capability to probe membrane protein conformations across various lipid environments.

SI MATERIALS AND METHODS

Expression, Purification and Labeling of BsYetJ

BsYetJ expression and purification were performed as previously described, with minor modifications.[1] A pET-24 derived pNYCOMPS vector containing a His-tag at the C-terminus of BsYetJ was used. Since wild-type BsYetJ is cysteine-free, this construct was directly used to prepare cysteine variants. Vectors with cysteine mutations at the indicated sites were generated using the QuikChange mutagenesis kit (Invitrogen), verified by DNA sequencing, and transformed into *E. coli* pLysS BL21(DE3) (Agilent) via heat shock. For BsYetJ expression, a dense 10 mL overnight culture was used to inoculate 0.5 L of TB media supplemented with 50 μg/mL kanamycin and 25 μg/mL chloramphenicol at 37 °C. Protein expression was induced with 0.4 mM isopropyl β-D-thiogalactopyranoside (IPTG) when the culture reached an OD_{600} of 0.6-0.8. After 4 h, cells were harvested by centrifugation at 7000g for 5 min at 4 °C and stored at −80 °C.

For BsYetJ purification, the cell pellets were suspended in lysis buffer (50 mM HEPES, 0.3 M NaCl, 20 mM imidazole, 5% glycerol, and 1 mM $MgCl_2$, pH 7.8) and sonicated. The lysate was cleared by centrifugation at 12800g for 30 min, and the resulting supernatant was subjected to a second centrifugation at 45000g for 1 h to obtain the membrane pellets. These pellets were solubilized in lysis buffer with an additional 1.5% (w/v) β-DDM at ambient temperature to extract membrane-bound BsYetJ. After 2 h, the lysate was filtered through a 0.45 μm Nylon filter to remove insoluble components and loaded onto a 5 mL HisTrap HP column (GE Healthcare). The column was washed with 20 column volumes (CV) of wash buffer (50 mM HEPES, 0.5 M NaCl, 75 mM imidazole, 5% (v/v) glycerol, and 0.05% (w/v) β-DDM, pH 7.8) and eluted with 5 CV of elution buffer (50 mM HEPES, 0.3 M NaCl, 0.5 M imidazole, 5% (v/v) glycerol, and 0.05% (w/v) β-DDM, pH 7.8). The purified BsYetJ was then buffer-exchanged into storage buffer (50 mM HEPES, 0.3 M NaCl, 2.5% (v/v) glycerol, and 0.05% (w/v) β-DDM, pH 7). The purity was confirmed by SDS-PAGE.

Fluorophore labeling was performed by adding a 10-fold molar excess of Alexa Fluor 647 C_2 maleimide or Alexa Fluor 568 C_5 maleimide (Thermo Scientific) from a 10 mM stock in DMSO.

The reaction was kept in the dark for 15 h at 4 °C, followed by buffer exchange to remove unbound fluorophore. Labeling efficiencies were calculated by measuring the UV-vis absorption spectra of Fluo-BsYetJ in detergent micelles using a U-3900 spectrometer (Hitachi) (Fig. S1A).

For ESR measurements, purified cysteine variants were spin-labeled with a 40-fold molar excess of MTSSL (1-oxyl-2,2,5,5-tetramethylpyrroline-3-methyl methanethiosulfonate) (Enzo Life Sciences) from a 0.1 M stock for 20 h at 4 °C. Following incubation, the reaction mixture was buffer-exchanged to remove free spin label, and the resulting spin-labeled BsYetJ was stored at -80 °C until nanodisc reconstitution.

Expression and Purification of Membrane Scaffold Protein

Membrane scaffold protein (MSP1D1, referred to as MSP for simplicity in this study) was expressed and purified as previously described, with minor modifications.[2] Briefly, a fresh colony of *E. coli* BL21(DE3) (Agilent) containing the MSP1D1 gene in pET-28a vector (Addgene) was transferred to TB media supplemented with 50 μg/mL of kanamycin and grown at 37 °C until OD_{600} reached to 0.6-0.8. Subsequently, 10 mL of this culture was inoculated in 0.5 L of TB at 37 °C, and MSP1D1 expression was induced with 1 mM IPTG when the culture OD_{600} reached 2.5. After 4 h, the cells were harvested by centrifugation at 7000g and stored at −80 °C.

For purification, the cell pellets were suspended in 20 mM sodium phosphate, 0.1 M NaCl, 1% (v/v) Triton X-100, 10 mM MgSO4, pH 7.4, with 10 μg/mL DNase I and 300 μL 0.1 M phenylmethylsulfonyl fluoride (PMSF) solution in ethanol, and then sonicated. Cell debris was removed by centrifugation at 12800g, and the lysate was filtrated through a 0.45 μm Nylon filter before being loaded onto a 5 mL HisTrap HP column. The column was washed with the following order of buffer: (i) 25 mL of 40 mM Tris-HCl, 0.3 M NaCl, and 1% (v/v) Triton X-100, pH 8.0 (ii) 25 mL of 40 mM Tris-HCl, 0.3 M NaCl, and 50 mM sodium cholate, pH 8.0 (iii) 40 mM Tris-HCl, 0.3 M NaCl, and 40 mM imidazole, pH 8.0. MSP1D1 was eluted with 40 mM Tris-HCl, 0.3 M NaCl, and 0.4 M imidazole, pH 8.0 and then buffer-exchanged into storage buffer (20 mM Tris-

HCl, 0.1 M NaCl, pH 7.4). Protein purity was checked by SDS-PAGE, and the purified MSP1D1 was concentrated and stored at −80 °C.

Reconstitution of BsYetJ into Nanodiscs

We followed previously established protocols to prepare BsYetJ-loaded nanodiscs samples by mixing Fluo-labeled or spin-labeled BsYetJ with MSP, lipid, and sodium cholate in the molar ratio of 1: 4: 240: 480.[1,3] Pure POPC lipid (1-palmitoyl-2-oleoyl- glycero-3-phosphocholine) (Avanti Polar Lipids) was used in this study. After a 30-min incubation on ice, Biobeads SM-2 (1 g/mL) (Bio-Rad) were added to the mixture and incubated overnight at 4 °C. The following day, Biobeads were removed and the nanodiscs were run on a Superdex 200 Increase 10/300 GL gel filtration column (GE Healthcare). Fractions containing nanodiscs were collected, concentrated, and stored at −80 °C until further usage. The incorporation rate of BsYetJ into nanodiscs was calculated by measuring the UV-Vis absorption of nanodiscs containing Fluo-BsYetJ, using the known fluorophore-labeling efficiency (Fig. S1A-B).

Nano-delivery Efficiency for *E. coli* Membranes

E. coli pLysS BL21(DE3) containing the pNYCOMPS vector, which encodes a stop codon prior to the BsYetJ sequence, was grown in TB media supplemented with 50 μg/mL of kanamycin and 25 μg/mL of chloramphenicol at 37 °C. When the OD_{600} reached approximately 1, 1 mL of the culture was collected, and the *E. coli* cells were pelleted by centrifugation. The cells were then suspended in 0.5 mL of *E. coli* buffer (50 mM Tris, 60 mM NaCl, 0.5% glucose, pH 7.5), followed by the addition of 0.75 μM Alexa-647-labeled BsYetJ nanodiscs to initiate protein delivery. Given that 1 mL of culture at $OD_{600} = 1$ contains roughly 8×10^8 cells, this corresponds to a molar ratio of approximately 3×10^5 between BsYetJ nanodiscs and *E. coli* cells.[4] BsYetJ insertion into *E. coli* membranes was proceeded at 37 °C for various incubation times (Fig. 2A) to study the kinetics of protein delivery. Based on the observed 40% transfer rate after a 1-h incubation (Fig. 2A), the nanodiscs successfully delivered approximately 1.2×10^5 molecules of BsYetJ to the membrane of each E. coli cell within 1 h.

Afterwards, the cells were washed three times with *E. coli* buffer and loaded into a black polystyrene 96-well plate (Thermo Scientific). Fluorescence emission intensities of Alexa-647 at 681 nm upon excitation at 651 nm were monitored using a Synergy H1 Microplate Reader device (BioteK), and the fluorescence from 0.5 mL of 0.75 µM Alexa-647-labeled BsYetJ nanodiscs was used as a reference to calculate delivery efficiency.

After fluorescence quantification, *E. coli* cells from 1-h nanodiscs incubation were retrieved and incubated with 10 µM PM-1 fluorescent dye[5] for 1 h. The resulting cells were washed multiple times with *E. coli* buffer and loaded into a µ-slide 18-well chamber (Ibidi GmbH). Images of *E. coli* cells were captured using a laser scanning confocal microscope (Zeiss LSM 700) with a 63× oil objective. Fluorescent images for Alexa-647 and PM-1 were taken using 639 nm and 405 nm lasers, respectively. The images were then processed by ZEN 2009 Light Edition software (Zeiss) and ImageJ.

Nano-delivery Efficiency for *B. subtilis* Membranes

Bacillus subtilis (ATCC 23857) containing pHT254 vector was grown in LB media supplemented with 5 µg/mL chloramphenicol at 37 °C until the OD_{600} reached approximately 1. For delivery efficiency to native *B. subtilis*, cells from 1 mL of the culture were collected and directly incubated with 0.75 µM Alexa-568-labeled BsYetJ nanodiscs in 0.5 mL of *B. subtilis* buffer (LB media with an additional 0.5 M sucrose and 20 mM $MgCl_2$) at 37 °C.

For the preparation of *B. subtilis* protoplasts, we followed previously published protocols and subjected the cells to 1 mg/mL lysozyme (Biotech) in *B. subtilis* buffer at 37 °C for 1 h.[6] The resulting protoplasts were then incubated with 0.75 µM Alexa-568-labeled BsYetJ nanodiscs at 37 °C for 1 h. After incubation, the cells were washed three times, and the fluorescence emission intensities of Alexa-568 at 610 nm upon excitation at 575 nm were measured.

To check the viability of the cells, part of the protoplasts after fluorescence measurements was diluted and plated on an LB agar plate. The remaining protoplasts underwent the same procedures used for *E. coli* cells to obtain confocal images, with the exceptions that *B. subtilis* buffer was used, and a 555 nm laser was used for Alexa-568 fluorescence excitation instead of a 639 nm laser.

In-cell Calcium Flux Assay

Calcium flux assays were performed in *E. coli* and *B. subtilis* cells following protocols previously reported with minor modifications.[7] The same *E. coli* and *B. subtilis* constructs used in the nano-delivery efficiency tests were cultivated at 37 °C until OD_{600} reached approximately 1. For *E. coli*, cells from 1 mL of the culture were collected by centrifugation and then incubated with 10 µM of the calcium indicator Fluo-8/AM (AAT Bioquest) in 0.5 mL of *E. coli* buffer supplemented with 200 µM EDTA at 37 °C. For *B. subtilis*, protoplasts were prepared using the cells from 1 mL of the culture, following the previously mentioned protocols. The protoplasts were then incubated with 10 µM of Fluo-8/AM in 0.5 mL of *B. subtilis* buffer containing 200 µM EDTA at 37 °C. Both *E. coli* and *B. subtilis* were incubated with Fluo-8/AM for 2 h. Afterwards, WT BsYetJ-loaded nanodiscs were added directly to the reaction mixtures with a final concentration of 0.75 µM and incubated at 37 °C for 1 h to facilitate BsYetJ insertion. The cells loaded with Fluo-8/AM and BsYetJ were washed twice with the corresponding buffer to remove any unloaded Fluo-8/AM and unincorporated nanodiscs. The resulting cells were harvested by centrifugation and were suspended in 0.6 mL of the corresponding buffer supplemented with 100 µM EDTA.

Calcium flux activity was initiated by the addition of 10 mM $CaCl_2$. Fluorescence emission intensities of Fluo-8/AM at 525 nm upon excitation at 490 nm were monitored using a Synergetic H1 Microplate Reader device (BioTeK). Intracellular free calcium concentration was determined using the formula $[Ca^{2+}]_{cyto} = K_d \times (F - F_{min})/(F_{max} - F)$, where K_d is 389 nM as recommended by the manufacturer. F_{min} represents the fluorescence intensity before the addition of calcium, F is the time-dependent fluorescence intensity in response to 10 mM $CaCl_2$, and F_{max} is the fluorescence intensity from calcium-saturated Fluo-8/AM, measured after the addition of 20 µL 10% Triton X-100 to ensure complete cell permeation. Together with our fluorescence-based and DEER

measurements, we clearly demonstrate that this nano-delivery method efficiently delivers large quantities of BsYetJ while preserving its structure and functionality (Fig. S3).

Continuous Wave (CW) ESR Measurements

Sample preparation for CW-ESR measurements was performed by incubating *E. coli* cells from 2.5 mL of OD_{600} = 1 culture with 65 µM BsYetJ-33R1-nanodiscs in 0.3 mL of *E. coli* buffer at 37 °C. After 1 h, the cells were washed three times to remove suspended nanodiscs and loaded into ESR capillary tubes with a sample volume of 25 µL. *E. coli* cells without nanodiscs incubation were used as a control. CW-ESR measurements were performed at X-band frequency (9.4 GHz) with the following parameters: 100 kHz modulation frequency, 0.1 G modulation amplitude, 10.24 ms time constant, 10.24 ms conversion time, 200 G sweep width, 1024 points, 15 scans and an incident microwave power of 1.5 mW using a Bruker ELEXSYS E580-400 CW/pulse spectrometer at 298 K. The viability of the *E. coli* cells after ESR measurements was confirmed by plating on LB agar plates.

Pulse ESR Measurements and Analysis

To prepare samples for in-cell DEER measurements, *E. coli* cells from a 12.5 mL culture at OD_{600} = 1 were collected by centrifugation and resuspended in 1.5 mL of *E. coli* buffer. Spin-labeled BsYetJ-loaded nanodiscs were added to a final concentration of 40 µM, and the mixture was incubated at 37 °C for 1 h for protein delivery. After incubation, the cells were washed three times with *E. coli* buffer containing 15% (v/v) glycerol to remove unincorporated nanodiscs. For *B. subtilis* samples, cells from a 15 mL culture at OD_{600} = 1 were prepared into protoplasts following standard protocols. The protoplasts were collected by centrifugation, resuspended in 2 mL of *B. subtilis* buffer, and incubated with spin-labeled BsYetJ-loaded nanodiscs at a final concentration of 30 µM at 37 °C for 1 h. After incubation, the protoplasts were washed three times with *B. subtilis* buffer containing 15% (v/v) glycerol to remove unincorporated nanodiscs.

For both *E. coli* and *B. subtilis* samples, the washed cells were pelleted, resuspended in their respective buffers with 15% (v/v) glycerol to a final volume of 40 μL, and transferred into ESR quartz tubes (i.d. 3 mm). This process concentrates the 30-40 μM solution in 1.5-2 mL to a final volume of 40 μL. With a transferring efficiency of 25-40% (Fig. 2) and a spin-labeling efficiency of 50-70%, the estimated concentration of spin-labeled BsYetJ in the ESR tube is approximately 250 μM. To prevent nitroxide reduction, the samples were plunge-frozen and stored in liquid nitrogen until DEER measurements.

Pulse ESR experiments were conducted on a Bruker ELEXSYS E580-400 X-band CW/pulse spectrometer with a split-ring resonator (EN4118X-MS3) equipped with a cryogenic ultralow-noise microwave amplifier and a helium gas flow system (4118CF and 4112HV) at 80 K.[8] In this cryoprobe head, spin echo signals are preamplified before entering the microwave bridge, significantly enhancing sensitivity and SNR, thus reducing data acquisition time.

For the electron spin echo (ESE) experiments, the Carr-Purcell sequence with two-step phase cycling was used. This sequence consists of a $\pi/2$ pulse along the x-axis followed by a delay τ and a train of π pulses, separated by interpulse delay 2τ. The pulse frequency was set to the maximum of the echo-detected field swept spectrum, with the $\pi/2$ and π pulse durations set to 16 and 32 ns, respectively.

For DEER measurements (X-band), we employed a dead-time free four-pulse constant-time DEER sequence with two-step phase cycling.[9] The durations of the observer π and $\pi/2$ pulses were set to 32 ns and 16 ns, respectively, with a frequency offset of 65 MHz above the pump pulse frequency. The pump pulse duration was approximately 32 ns and was positioned at the maximum of the echo-detected field-swept spectrum. All pulses were amplified using a pulsed traveling wave tube (TWT) amplifier (E580-1030), and each set of data was accumulated over 15-20 minutes. Data analysis of the DEER measurements was conducted as follows. Initially, background signals in the time-domain DEER traces were removed using the DeerAnalysis program, followed by Tikhonov regularization to obtain model-free distance distributions.[10,11] The dimensionality of the DEER

data ranged from 2.3 to 2.5, typical for homogeneously distributed spin-labeled molecules reconstituted in membrane vesicles. These model-free results were further analyzed and modeled as a sum of Gaussians using established methods.[12] Model-based analysis and error estimations were performed with the DD software (version 7).[12] Additionally, the DEERNet software, a deep neural network for one-step DEER data analysis, was used to provide an independent assessment.[9,13] The distance distributions obtained from both the model fits (Fig. 3B) and DEERNet (Fig. S6) were consistent with each other, confirming the reliability of the results.

A Note on the Comparison of In-Situ Labeling and Nano-Delivery Methods

There are fundamental differences between the in-situ labeling method and the nano-delivery approach described in this study, each with its own advantages and limitations. In the nano-delivery method, membrane proteins (MPs) are spin-labeled before incorporation into nanodiscs. These MP-loaded nanodiscs are then incubated with living cells, transferring the MPs to the cell membranes. This method offers versatility in labeling positions, not restricted to surface-exposed residues, facilitating DEER measurements in diverse cellular and lipid environments. Although it does not ensure uniform orientation of the delivered MPs, this approach can effectively enhance the SNR of DEER data. Additionally, it allows the study of the same MP in different cells, thus exploring various lipid environments.

In contrast, in-situ labeling is performed directly in the cellular environment, which is highly reactive to reducing spin probes, posing challenges for sample preparation. This reduction challenge is being addressed through the development of more robust spin labels and optimized delivery techniques.[14–17] The in-situ labeling method restricts labeling to solvent-exposed residues on the extracellular side but ensures the correct orientation of MPs in *E. coli* cells. Using isolated outer membrane preparations allows access to both membrane surfaces for spin labeling and ligand binding, though the orientations of MPs are not uniform.

Overall, the choice between these methods depends on the specific research questions and proteins under investigation. Both methods represent significant advancements in the in-cell study of membrane proteins using DEER spectroscopy, each contributing valuable insights into the structural dynamics of proteins in their native environments.

REFERENCES

(1) Li, C.-C.; Kao, T.-Y.; Cheng, C.-C.; Chiang, Y.-W. Structure and Regulation of the BsYetJ Calcium Channel in Lipid Nanodiscs. *Proc Natl Acad Sci USA* **2020**, *117* (48), 30126–30134.

(2) Yeh, P.-S.; Li, C.-C.; Lu, Y.-S.; Chiang, Y.-W. Structural Insights into the Binding and Degradation Mechanisms of Protoporphyrin IX by the Translocator Protein TSPO. *JACS Au* **2023**, *3* (10), 2918–2929.

(3) Li, C.-C.; Hung, C.-L.; Yeh, P.-S.; Li, C.-E.; Chiang, Y.-W. Doubly Spin-Labeled Nanodiscs to Improve Structural Determination of Membrane Proteins by ESR. *RSC Adv.* **2019**, *9* (16), 9014–9021.

(4) Mira, P.; Yeh, P.; Hall, B. G. Estimating Microbial Population Data from Optical Density. *PLoS ONE* **2022**, *17* (10), e0276040.

(5) Xu, S.-Q.; Sie, Z.-Y.; Hsu, J.-I.; Tan, K.-T. Small Plasma Membrane-Targeted Fluorescent Dye for Long-Time Imaging and Protein Degradation Analyses. *Anal. Chem.* **2023**, *95* (42), 15549–15555.

(6) Marcone, G. L.; Carrano, L.; Marinelli, F.; Beltrametti, F. Protoplast Preparation and Reversion to the Normal Filamentous Growth in Antibiotic-Producing Uncommon Actinomycetes. *J Antibiot* **2010**, *63* (2), 83–88.

(7) Chang, Y.; Bruni, R.; Kloss, B.; Assur, Z.; Kloppmann, E.; Rost, B.; Hendrickson, W. A.; Liu, Q. Structural Basis for a pH-Sensitive Calcium Leak across Membranes. *Science* **2014**, *344* (6188), 1131–1135.

(8) Šimėnas, M.; O'Sullivan, J.; Zollitsch, C. W.; Kennedy, O.; Seif-Eddine, M.; Ritsch, I.; Hülsmann, M.; Qi, M.; Godt, A.; Roessler, M. M.; Jeschke, G.; Morton, J. J. L. A Sensitivity Leap for X-Band EPR Using a Probehead with a Cryogenic Preamplifier. *Journal of Magnetic Resonance* **2021**, *322*, 106876.

(9) Schiemann, O.; Heubach, C. A.; Abdullin, D.; Ackermann, K.; Azarkh, M.; Bagryanskaya, E. G.; Drescher, M.; Endeward, B.; Freed, J. H.; Galazzo, L.; Goldfarb, D.; Hett, T.; Esteban Hofer, L.; Fábregas Ibáñez, L.; Hustedt, E. J.; Kucher, S.; Kuprov, I.; Lovett, J. E.; Meyer, A.; Ruthstein, S.; Saxena, S.; Stoll, S.; Timmel, C. R.; Di Valentin, M.; Mchaourab, H. S.; Prisner, T. F.; Bode, B. E.; Bordignon, E.; Bennati, M.; Jeschke, G. Benchmark Test and Guidelines for DEER/PELDOR Experiments on Nitroxide-Labeled Biomolecules. *J. Am. Chem. Soc.* **2021**, *143* (43), 17875–17890.

(10) Chiang, Y.-W.; Borbat, P. P.; Freed, J. H. The Determination of Pair Distance Distributions by Pulsed ESR Using Tikhonov Regularization. *J. Magn. Reson.* **2005**, *172* (2), 279–295.

(11) Jeschke, G.; Chechik, V.; Ionita, P.; Godt, A.; Zimmermann, H.; Banham, J.; Timmel, C. R.; Hilger, D.; Jung, H. DeerAnalysis2006—a Comprehensive Software Package for Analyzing Pulsed ELDOR Data. *Appl. Magn. Reson.* **2006**, *30* (3–4), 473–498.

(12) Stein, R. A.; Beth, A. H.; Hustedt, E. J. A Straightforward Approach to the Analysis of Double Electron–Electron Resonance Data. In *Methods in Enzymology*; 2015; pp 531–567.
(13) Keeley, J.; Choudhury, T.; Galazzo, L.; Bordignon, E.; Feintuch, A.; Goldfarb, D.; Russell, H.; Taylor, M. J.; Lovett, J. E.; Eggeling, A.; Fábregas Ibáñez, L.; Keller, K.; Yulikov, M.; Jeschke, G.; Kuprov, I. Neural Networks in Pulsed Dipolar Spectroscopy: A Practical Guide. *Journal of Magnetic Resonance* **2022**, *338*, 107186.
(14) Goldfarb, D. Exploring Protein Conformations in Vitro and in Cell with EPR Distance Measurements. *Current Opinion in Structural Biology* **2022**, *75*, 102398.
(15) Ketter, S.; Gopinath, A.; Rogozhnikova, O.; Trukhin, D.; Tormyshev, V. M.; Bagryanskaya, E. G.; Joseph, B. In Situ Labeling and Distance Measurements of Membrane Proteins in *E. Coli* Using Finland and OX063 Trityl Labels. *Chemistry A European J* **2021**, *27* (7), 2299–2304.
(16) Ketter, S.; Dajka, M.; Rogozhnikova, O.; Dobrynin, S. A.; Tormyshev, V. M.; Bagryanskaya, E. G.; Joseph, B. In Situ Distance Measurements in a Membrane Transporter Using Maleimide Functionalized Orthogonal Spin Labels and 5-Pulse Electron-Electron Double Resonance Spectroscopy. *Journal of Magnetic Resonance Open* **2022**, *10–11*, 100041.
(17) Joseph, B.; Jaumann, E. A.; Sikora, A.; Barth, K.; Prisner, T. F.; Cafiso, D. S. In Situ Observation of Conformational Dynamics and Protein Ligand–Substrate Interactions in Outer-Membrane Proteins with DEER/PELDOR Spectroscopy. *Nat Protoc* **2019**, *14* (8), 2344–2369.
